# Lightweight Fan-Beam Microstrip Grid Antenna for Airborne Microwave Interferometric Radiometer Applications

**DOI:** 10.3390/mi14010228

**Published:** 2023-01-15

**Authors:** Chunwang Gu, Hao Liu, Min Yi

**Affiliations:** 1 Key Laboratory of Microwave Remote Sensing, National Space Science Center, Chinese Academy of Sciences, Beijing 100190, China; 2 University of Chinese Academy of Sciences, Beijing 100049, China

**Keywords:** microwave interferometric radiometer, microstrip grid antenna, sparse array

## Abstract

The microwave interferometric radiometer (MIR) uses aperture synthesis technology to equate multiple small-aperture antennas into a large-aperture antenna to improve spatial resolution. At present, MIR antennas that operate at frequencies above the C-band mostly use horn antennas, waveguide slot antennas, etc., which have the disadvantages of a high profile and large mass. In this paper, a new type of miniaturized, low-profile, and lightweight K-band fan-beam microstrip grid antenna is designed for the airborne campaign of the K-band one-dimensional MIR of a Microwave Imager Combined Active and Passive (MICAP) onboard a Chinese Ocean Salinity Mission (COSM). With a limited size constraint (12.33 mm) on the antenna width, a fan-beam shape antenna pattern was achieved with a 5.34° 3-dB beamwidth in the narrow beam direction and up to a 55° 3-dB beamwidth in the fan-beam direction. A periodic structural unit is proposed in this paper to reduce the design complexity of Taylor weighting, achieving desirable performances on gain (19.1 dB) and sidelobe level (<−20 dB) in the H-plane. Four antenna elements were fabricated and arranged in a non-redundant sparse array. The performance of the four-element sparse array was evaluated with a simulation and real measurement in an anechoic chamber. The coupling between antenna elements was less than −25 dB, and the consistency of phase patterns was better than 3.4°. These results verify the feasibility of the proposed K-band microstrip grid antenna for airborne MIR applications.

## 1. Introduction

The microwave interferometric radiometer (MIR), also called a synthetic aperture radiometer, is a passive microwave measuring instrument with a high resolution, which has great application potential in the field of microwave remote sensing [1]. MIRs use aperture synthesis technology to equate multiple small-aperture antennas to a large-aperture antenna for improving spatial resolution. In order to meet the requirement of the shortest baseline length of the synthetic aperture sparse array (normally half a wavelength or slightly larger, according to the field-of-view requirement), the MIR has strict constraints on the size of the unit antenna, that is, the size of the antenna should be less than the shortest baseline length. For the fan-beam antenna of a one-dimensional MIR (1D MIR), the narrow side size of the antenna is less than the shortest baseline length.

Generally, the antenna forms used in MIR design include the microstrip patch antenna [2,3,4,5,6], the waveguide slot antenna [7,8], the parabolic cylindrical antenna [9,10], and the horn antenna [11,12,13]. For a low-frequency band up to a C-band, there is normally enough of a dimensional margin to design a patch antenna to meet the minimum baseline length requirement of the synthetic aperture array. Therefore, currently, patch antennas are used in several 1D and 2D MIR systems, i.e., SMOS/MIRAS in L-band (1.4 G) [2], FPIR in L-band(1.4 GHz) [6], and HIRAD in C-band(6.9 GHz) [5]. When the detection band of the MIR is higher than a C-band, due to the shorter wavelength of the detection band, the design of the microstrip antenna has challenges under the limitation of the shortest baseline for the synthetic aperture array. In this case, MIRs mostly use technically mature horn antennas and parabolic cylinder reflector antennas with the horn as the feed source. Obviously, these antennas have the disadvantages of a high profile, heavy mass, and difficult integration. The grid antenna was proposed by John Kraus in 1964 [14]. After long-term theoretical development [15,16], grid antennas with characteristics of a compact structure, convenient feeding, and easy integration are now widely used in miniaturized vehicle radar [17,18,19,20,21], MIMO systems [22], and 5G communication [23,24].

Microwave Imager Combined Active and Passive (MICAP) is a new type of multi-frequency active/passive payload proposed for a COSM satellite. MICAP consists of three 1D MIRs working at the L/C/K band (1.4/6.9/18.7 GHz) and a digital beamforming scatterometer at the L-band (1.25 GHz), sharing a large 3 m × 5.5 m parabolic cylinder reflector antenna with a line array feed [25]. An airborne campaign of MICAP was planned in a coastal area for the purpose of instrument performance testing and demonstration. An airborne prototype of a MICAP instrument is developed with a reduced aperture size and receiving units, and more importantly, with an alternative compact planar antenna that is suitable for an airborne platform.

Aiming at the application requirements of a MICAP airborne prototype, a miniaturized, low-profile, and lightweight fan-beam microstrip grid antenna at the K-band (18.7 GHz) is designed. First, a type of periodic structural unit is proposed in the design, which effectively reduces the computational complexity, enabling the antenna to include more radiating cells with Taylor weighting to achieve a higher gain, a narrower 3-dB beamwidth of the narrow beam, and a lower sidelobe level (SLL). Second, under the restriction that the minimum baseline of MICAP’s K-band 1D MIR airborne prototype is 0.8λ (12.83 mm), the proposed antenna has a wider 3-dB beamwidth of up to 55° of the wide beam, which meets the swath requirement of the MIR. Third, the proposed antennas are formed into a non-redundant sparse array of 1D synthetic aperture and analyzed. In the sparse array, the coupling of two antenna elements with a minimum geometric center distance (0.8λ) is less than −25 dB.

## 2. Design and Simulation of Microstrip Grid Antenna

In this paper, the center frequency of the fan-beam microstrip grid antenna is 18.7 GHz. The bandwidth of the antenna designed in this paper is 300 MHz, and the operating frequency band range is 18.55–18.85 GHz. The requirements for the fan beam include the following: the 3-dB beamwidth in the narrow beam direction (H-plane, Along-Track) should be less than 6° (determined by the resolution in the narrow beam direction), and the 3-dB beamwidth in the wide beam direction (E plane, Cross-Track) should be greater than 55° (determined by the swath of the MIR). For radar and MIMO systems, the size of the antennas is not overly stringent. However, for the 1D MIR to meet the minimum baseline length of 0.8λ for forming a synthetic aperture sparse array in the wide beam direction, the narrow edge width of the microstrip grid antenna must be less than 0.8λ (0.8λ ≈ 12.83 mm). A narrow edge width of the antenna that is too small will affect the 3-dB beamwidth in the wide beam direction. It is important to design the microstrip grid antenna with a wide beamwidth in this direction under the restriction of a narrow edge width.

In the structure of the a typical microstrip grid antenna, the short side length of the grid is $s=\frac{\lambda_{g}}{2}\;,$ and the long side length of grid is $l=\lambda_{g}$, where $\lambda_{g}=\lambda /\sqrt{\varepsilon_{r}}$ is the Waveguide wavelength. According to the current distribution of the grid antenna [15,26], the current on the long side of the grid is out of phase, and the current on the short side is in phase, which results in the radiation formed on the long side cancelling itself out and only the short side producing radiation, thus reducing the cross-polarization level of the antenna. The long side of the grid is used as the transmission line, and the short side is used as the radiating cell.

In order to ensure the narrower 3-dB beamwidth in the narrow beam direction, a sufficient number of radiation cells are needed. At the same time, the pattern in this direction should have a lower sidelobe level.

**Figure 1:**
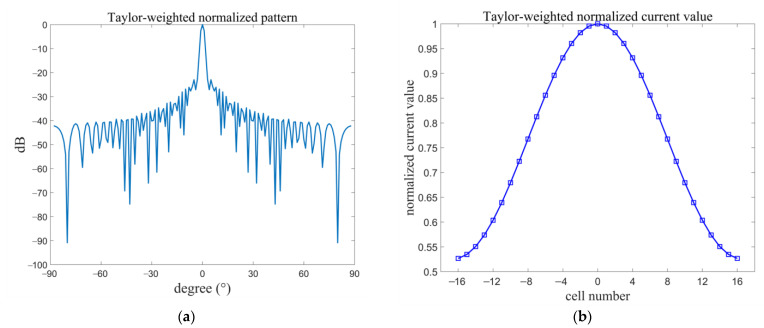
(**a**) Pattern of 33-cell array antenna by Taylor synthesis; (**b**) current distribution of every cell by Taylor synthesis.

The grid antenna designed in this paper is regarded as a linear array. The Taylor synthesis method is used to change the current distribution of the microstrip grid antenna, so as to achieve the requirement that the sidelobe level is less than −20 dB. In order to allow for a margin for the fabricated antenna, the sidelobe level value of the antenna was set to less than −25 dB in the process of designing the antenna. Through the programming calculation, a pattern of an array of 33 radiating cells in the antenna after Taylor synthesis as well as the current distribution of every cell are obtained, as shown in Figure 1. The half-power beamwidth of the pattern with the Taylor synthesis method is less than 6°, and the sidelobe level is less −25 dB.

**Figure 2:**
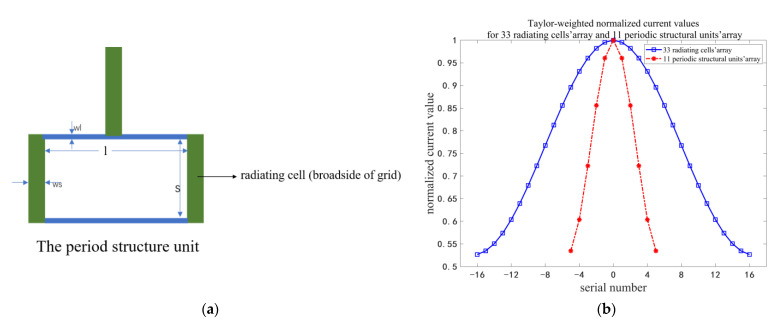
(**a**) The periodic structural unit; (**b**) Taylor-weighted normalized current distributions for 33 radiating cells’ array and 11 periodic structural units’ array.

**Figure 3:**
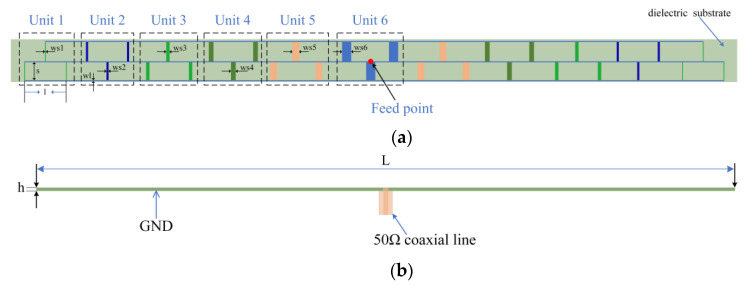
(**a**) Structure of the microstrip grid antenna; (**b**) the feeding structure.

The proposed periodic structural unit is shown in Figure 2a, which consists of three radiating cells. By arranging the periodic structural units and adding transmission lines, the microstrip grid antenna is formed, as shown in Figure 3a. Too many radiating cells will lead to an increase in the weighted parameters and improve the design complexity of the antenna. The Taylor-weighted current distribution of the 33 radiating cells’ array and the 11 periodic structural units’ array are shown in Figure 2b. The weighted parameters of the 33 radiation cells’ array are many. Through the analysis in Figure 2b, it is found that every three excitation current values of the 33 cells’ array are divided into one group, and the average current value of every group is approximately equal to the corresponding excitation current value of 11 periodic structural units’ array by Taylor weighting. For the periodic structural unit, the widths of the three radiating cells (broadside of grid) are set to the same value. The grid antenna with 33 radiating cells can be regarded as the array composed of 11 periodic structural units. The Taylor weighting for the 33 radiating cells can be converted into Taylor weighting for 11 periodic structural units in the grid antenna, which reduces the complexity of the antenna design and also ensures that the sidelobe level is <−25 dB. In addition, by using the periodic structural unit, the grid antenna still has a sufficient number of radiating cells to simultaneously ensure that the antenna has a high gain and a narrow half-power beamwidth.

The structure of the microstrip grid antenna is symmetrical and is shown in Figure 3a. The substrate is Rogers RT5880 with a relative dielectric constant of 2.2, a loss tangent of 0.0009, and a thickness of 0.787 mm. In this paper, the periodic structural units in the antenna are connected in a series. The ratio of the weighted current for 11 periodic structural units can be treated as the inverse ratio of the resistance. The characteristic impedance of the radiating cells for the periodic structural unit 6 is 50 Ω. The designed antenna is fed by a 50 Ω coaxial line inserted directly into the geometric center of the array, which makes the grid antenna form a non-traveling wave array to produce a stable beam and also lowers the complexity of the antenna feed structure, thus reducing the loss of electromagnetic waves. The feeding structure is shown in Figure 3b. The geometric dimensions of the antenna are listed in Table 1.

In the microstrip grid antenna, theoretical normalized current values calculated by the Taylor weighting for the 11 periodic structural units and the simulated normalized current values are shown in Table 2. These simulated current values are obtained by integrating the current distribution on the surface of each periodic structural unit in high-frequency electromagnetic simulation software. As can be seen from the Table 2, there are considerable differences between theoretical and simulated normalized current values for the periodic structural unit 1 and 2, but from the simulation results of the pattern and voltage standing wave ratio (VSWR) of the antenna in the following, the differences do not affect the overall performance of the antenna. The causes of the differences were analyzed: the size of unit 1 and 2 is small, resulting in their smaller current distribution. Moreover, the transmission line of the antenna in this paper is narrow, and the current fed to the distal units is reduced due to the transmission line loss.

**Figure 4:**
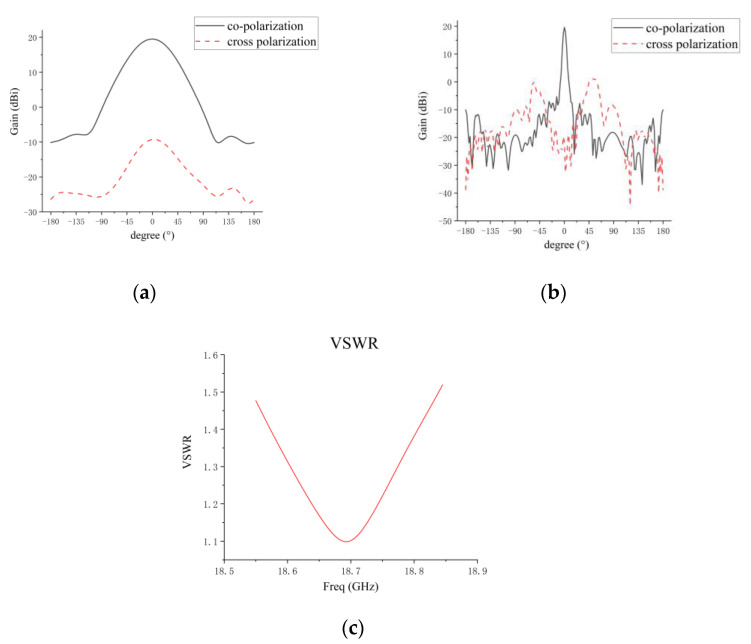
(**a**) Simulated results of E-plane pattern; (**b**) simulated results of H-plane pattern; (**c**) simulated results of VSWR.

The simulated results of the microstrip grid antenna are shown in Figure 4. The simulated gain of the microstrip grid antenna is 19.4 dBi. The simulation result of the E-plane pattern is presented in Figure 4a. The 3-dB beamwidth of the E-plane pattern is 61°, and the cross-polarization level is less than −28 dB. Figure 4b shows the simulation result of the H-plane pattern. The sidelobe level of the antenna is less than −25 dB, and the 3-dB beamwidth is 4.7°. The cross-polarization level is less than −18.7 dB. The simulation result of the VSWR is shown in Figure 4c. In the working frequency band of 18.55–18.85 GHz, the VSWR is less than 1.53. At the center frequency of 18.7 GHz, the VSWR is 1.12.

## 3. Simulation and Analysis of 1D Synthetic Aperture Non-Redundant Sparse Array Composed of Microstrip Grid Antenna

**Figure 5:**
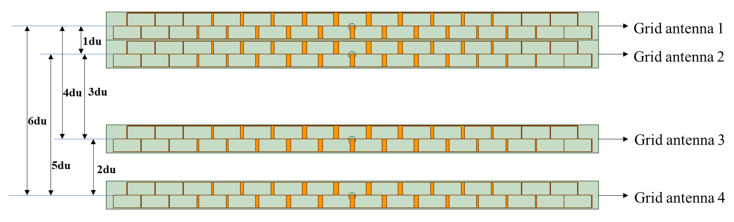
One-dimensional non-redundant sparse array composed of four microstrip grid antennas.

Figure 5 shows a one-dimensional non-redundant sparse array of the synthetic aperture composed of four microstrip grid antennas, which can generate continuous sampling baselines from 1 du to 6 du, where 1 du = 0.8λ (≈12.83 mm). For the sparse array, the performance of the coupling between the grid antennas, the VSWR, the amplitude, and the phase pattern of every antenna in the array environment are mainly analyzed.

**Figure 6:**
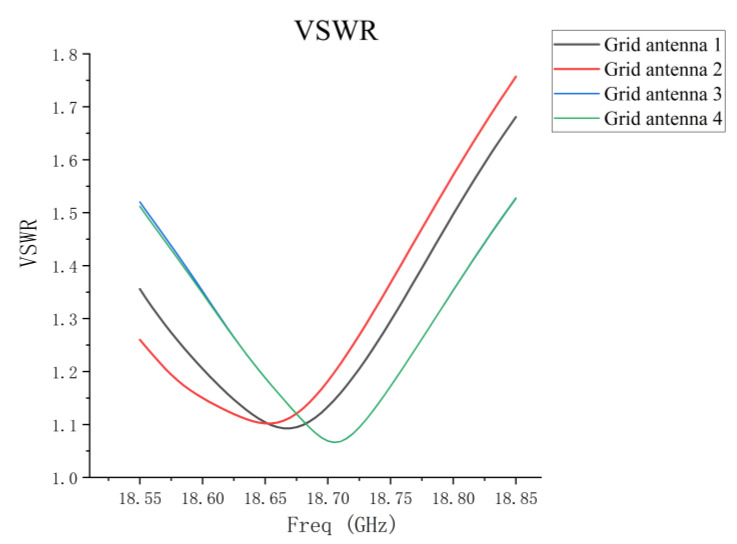
Simulated VSWR of four grid antennas in one-dimensional non-redundant sparse array.

The simulated VSWR for four microstrip grid antennas in the sparse array is presented in Figure 6. It can be seen that the resonant point of grid antenna 1 is shifted to the left by 25 MHz compared with the center frequency (18.7 GHz), and the maximum VSWR in the operating band is 1.69, which is deteriorated by 0.16 compared with the maximum VSWR of 1.53 in the simulation of the single grid antenna. In the case of minimum spacing (1 du) in the sparse array, the resonance point and the VSWR of grid antenna 1 are influenced by the coupling of the other antennas. The resonant frequency of grid antenna 2 is shifted to the left by 50 MHz, and the maximum VSWR is 1.76. The deterioration of grid antenna 2 is severer compared with grid antenna 1. The reason for this is that grid antenna 2 has a closer distance between antenna 3 and 4 than grid antenna 1. Grid antenna 3 and 4 are less affected by the coupling, and their VSWR simulation results are in good agreement with the single grid antenna.

**Figure 7:**
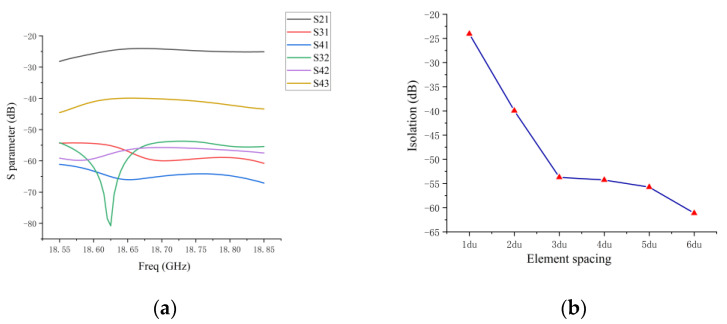
(**a**) Simulated coupling between antenna elements in sparse array; (**b**) the maximum values of simulated coupling between antenna elements with different du of sparse array in operating frequency band.

The simulation results of the coupling for every grid antenna in the 1D non-redundant sparse array are shown in Figure 7. The coupling value between antenna 1 and 2 in the sparse array is −24.9 dB, the coupling value between antenna 3 and 4 is −39.5 dB, and the coupling between the remaining antennas is less than −50 dB. It can be found that the coupling between the elements of the 1D non-redundant sparse array decreases with the increase in the baseline length formed by the antenna pairs.

**Figure 8:**
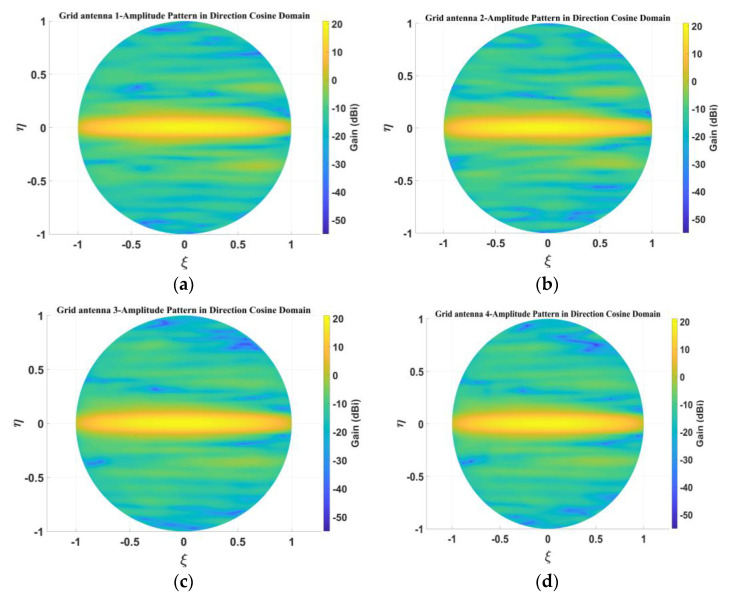
Simulated amplitude pattern in direction cosine domain: (**a**) grid antenna 1; (**b**) grid antenna 2; (**c**) grid antenna 3; (**d**) grid antenna 4.

**Figure 9:**
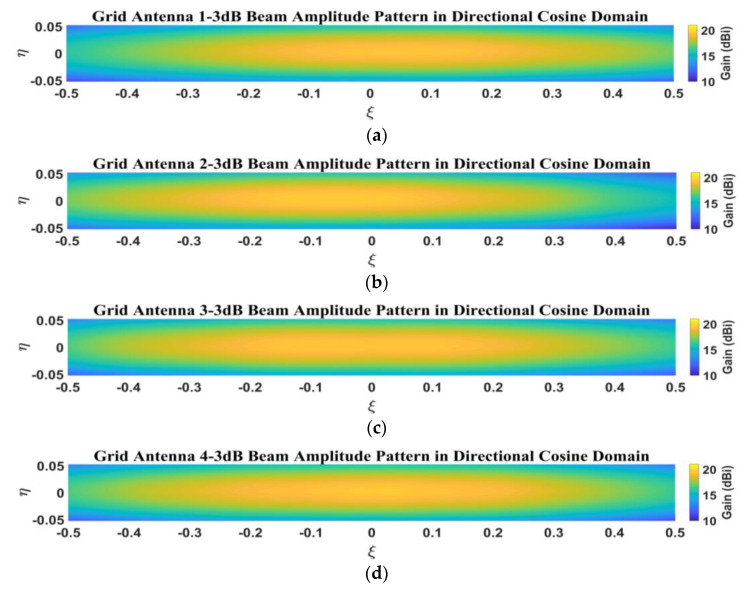
Simulated amplitude pattern of 3-dB beamwidth in direction cosine domain: (**a**) grid antenna 1; (**b**) grid antenna 2; (**c**) grid antenna 3; (**d**) grid antenna 4.

The inversion of the brightness temperature for the MIR needs to obtain the amplitude and phase patterns of the antenna in the direction cosine domain. The simulated amplitude patterns of the four elements for the 1D sparse array in the direction cosine domain are shown in Figure 8, and the amplitude patterns in the 3-dB beam range are shown in Figure 9. From Figure 8 and Figure 9, it can clearly be seen that in the sparse array environment, the amplitude patterns of the four elements are basically the same.

**Figure 10:**
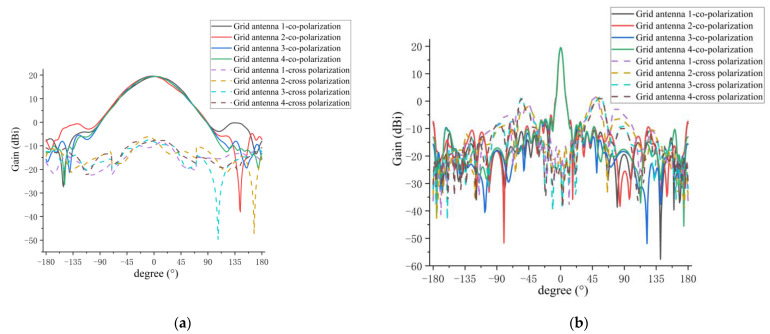
Simulated amplitude patterns for every antenna element in sparse array: (**a**) E-plane patterns; (**b**) H-plane patterns.

Figure 10 shows the amplitude patterns of the E and H planes for the four antenna elements in the sparse array. It can be seen from Figure 10a that the curves of the E-plane patterns for antenna 1 to 4 are basically the same within 3-dB beamwidth (±30°). The amplitude patterns of antennas 1 and 2 fluctuate to a certain extent around the angle of ±135°, indicating that the coupling effect between grid antenna 1 and antenna 2 is approximately symmetrical, resulting in a distortion of the pattern near the symmetrical angle. From Figure 10b, it can be seen that the curves of the co-polarization patterns in the H plane from grid antennas 1 to 4 are very consistent, and the cross-polarization level of every grid antenna in the H plane is also less than −17 dB, which is more consistent with the simulation of the cross-polarization level of the single grid antenna in the H plane.

**Figure 11:**
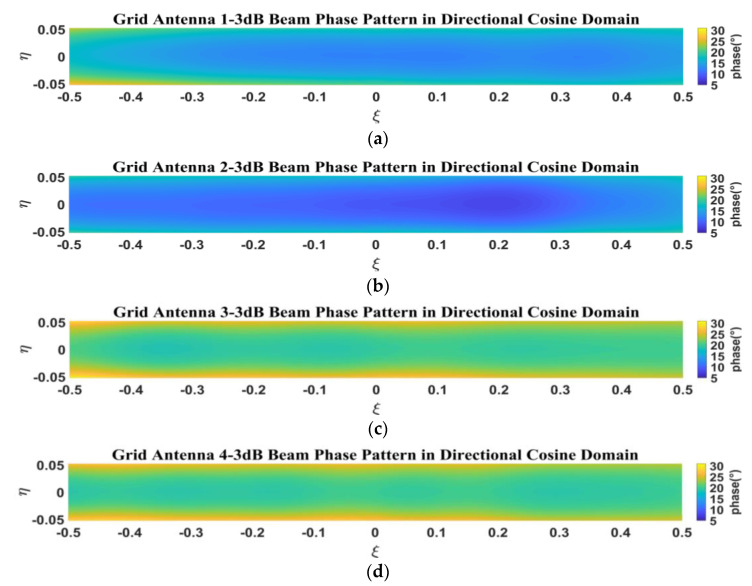
Simulated phase pattern of 3-dB beamwidth in direction cosine domain: (**a**) grid antenna 1; (**b**) grid antenna 2; (**c**) grid antenna 3; (**d**) grid antenna 4.

Figure 11 presents the simulated phase patterns within the 3-dB beamwidth for the four antenna elements in the sparse array. In Figure 11, the phase ranges of the phase patterns within the 3-dB beamwidth for grid antennas 1 and 2 are relatively consistent, and the phase ranges of antennas 3 and 4 are relatively consistent. This is due to the fact that antennas 1 and 2 are coupled more in the array environment, and antennas 3 and 4 are coupled less, resulting in the difference in phase consistency between them.

**Figure 12:**
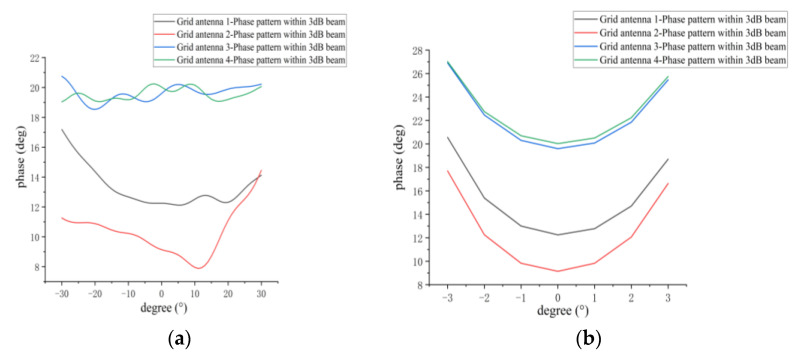
Simulated phase pattern of 3-dB beamwidth for every antenna element in sparse array: (**a**) E-plane phase pattern; (**b**) H-plane phase pattern.

Figure 12 shows the simulated phase patterns of the E and H planes within the 3-dB beamwidth for the four antenna elements in the 1D non-redundant sparse array. Table 3 gives the simulated phase ranges and phase fluctuations for the E and H planes in the 3-dB beamwidth range. It can be found that the phase fluctuations of the phase patterns in the wide beam direction (E plane) are smaller than those for the narrow beam direction (H plane). In both the E and H planes, the phase range of grid antennas 1 and 2 is consistent, and the phase range of antennas 3 and 4 is consistent, which is more consistent with the analysis of Figure 11. In addition, the phase fluctuations of grid antennas 1 and 2 are larger than antennas 3 and 4 due to the influence of coupling.

## 4. Measurement of the Designed Microstrip Grid Antenna

**Figure 13:**
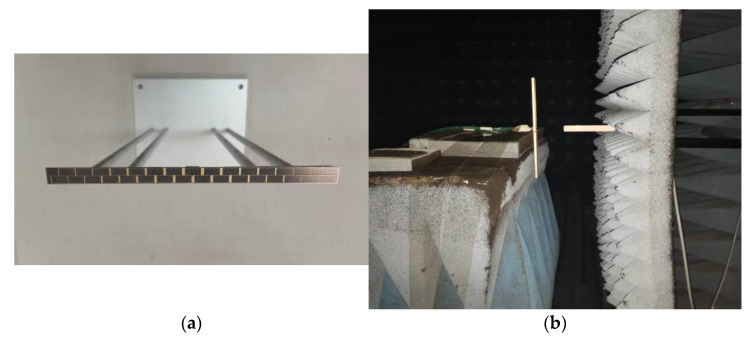
(**a**) Photograph of the fabricated grid antenna; (**b**) measurement of grid antenna in the anechoic chamber.

The fabricated microstrip grid antenna is shown in Figure 13a. The NSI2000 planar near-field measurement system was used to test the antenna pattern in the anechoic chamber. The test scene of the antenna is shown in Figure 13b.

**Figure 14:**
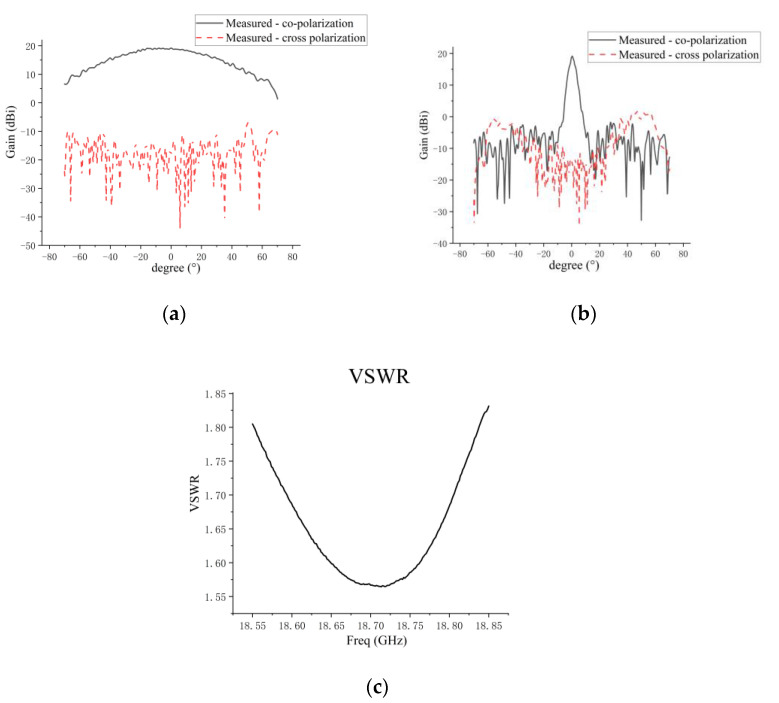
(**a**) Measured E-plane pattern of microstrip grid antenna; (**b**) measured H-plane pattern of microstrip grid antenna; (**c**) measured VSWR.

Figure 14 presents the measured results. The measured angle range of the antenna is ±70° for the planar near-field measurement system used in this paper. The measured patterns of the co-polarization and cross-polarization for the E plane and the H plane are shown in Figure 14a,b. The measured VSWR is presented in Figure 14c. The comparison results of the microstrip grid antenna simulation and measurement are given in Table 4. The measured VSWR of the antenna has deteriorated by about 0.3~0.4 in the whole frequency band compared with the simulated result. This deterioration is due to the processing error of the microstrip line limited in the narrow edge size (12.33 mm). In addition, the difference between the εr of the dielectric substrate for antenna processing and the theoretical value leads to the deterioration of the measured VSWR. It can be seen that ripples are produced in the E-plane pattern, which is due to the truncation error of the near–far field transformation in the planer near-field test system, so that result of the measured pattern has ripples.

## 5. Measurement and Analysis of 1D Synthetic Aperture Non-Redundant Sparse Array Composed of Microstrip Grid Antenna

### 5.1. VSWR and Coupling of 1D Non-Redundant Sparse Array

**Figure 15:**
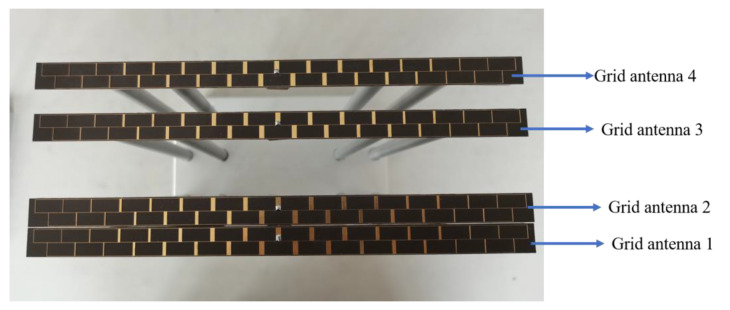
Photograph of the fabricated one-dimensional sparse array by microstrip grid antennas.

**Figure 16:**
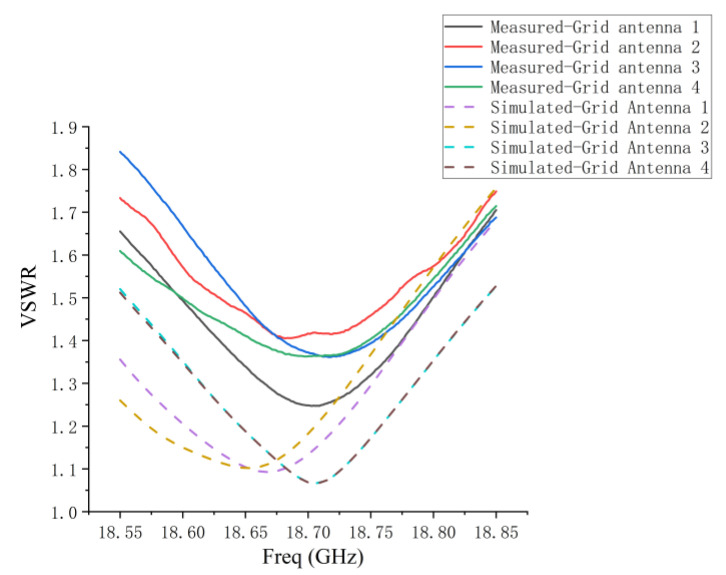
Comparison of measured and simulated VSWR for every antenna element in sparse array.

Figure 15 presents the fabricated one-dimensional non-redundant sparse array composed of four microstrip grid antennas. The dimensions of the sparse array are 223.6 mm × 89.3 mm. In the sparse array, a comparison between the measured and simulated results of the VSWR for every antenna element is shown in Figure 16. In the 1D non-redundant sparse array, the maximum measured VSWR of the antenna elements is less than 1.85. Compared with the simulation result, the increase in the measured VSWR of every antenna element in the array is due to the microstrip line processing error of each antenna element and the difference between εr of the dielectric substrate and the theoretical value, which is consistent with the analysis of the single grid antenna. In addition, compared with the measured VSWR of the single grid antenna in Figure 14c, the influence of the coupling between array elements makes the measured VSWR of the antenna elements in the array produce a small variation.

**Figure 17:**
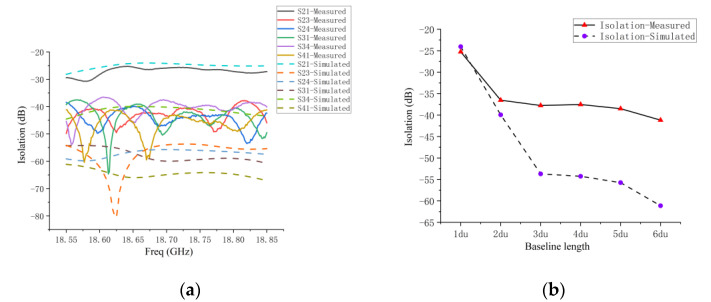
(**a**) Comparison of measured and simulated coupling between antenna elements in sparse array; (**b**) comparison of measured and simulated maximum coupling of antenna elements with different baseline lengths (du) in sparse array within operating frequency band.

The measured and simulated results of the coupling between the array elements are shown in Figure 17a. The maximum value of the measured coupling between antenna 1 and antenna 2 is −25 dB, which is very consistent with the simulated results. The measured coupling between the remaining antennas is higher than the simulation results. Figure 17b reflects the measured and simulated maximum coupling between antenna elements with a different baseline length (du) in the sparse array within the operating frequency band. The maximum and minimum measured coupling are −25 dB and −40 dB. Starting from the 3 du baseline, the measured coupling results differ from the simulated results, but the measured coupling of the antenna array with 2 du spacing (coupling of antenna 3 and 4) is already less than −35 dB, which is a very small coupling value. The difference between the measured and simulated results is due to the fact that the coupling in the actual antenna array environment is more complex than the simulated environment.

### 5.2. Measurement of Amplitude Patterns for 1D Non-Redundant Sparse Array

**Figure 18:**
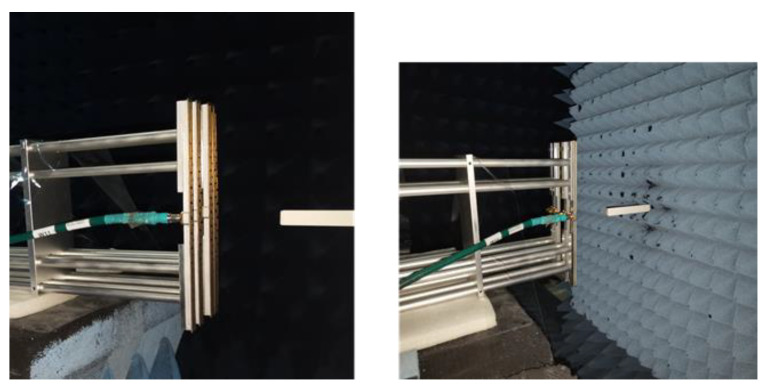
Measurement of fabricated one-dimensional sparse array in the anechoic chamber.

The NSI2000 planar near-field measurement system was used to measure the fabricated 1D non-redundant sparse array, and the test scene is shown in Figure 18. The test process is to feed each antenna in the sparse array separately, and the remaining antennas are connected to the matching load (in the system of the 1D MIR, each antenna element in the sparse array is connected to the corresponding receiver).

**Figure 19:**
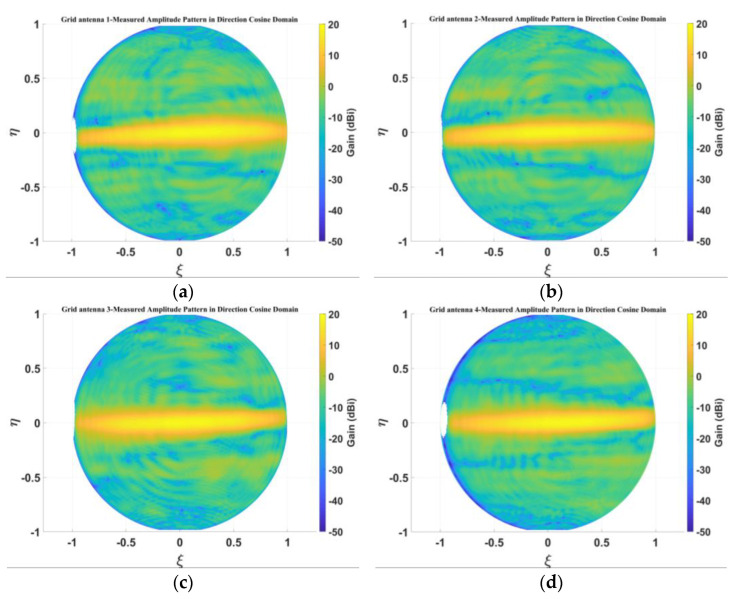
Measured amplitude pattern in direction cosine domain: (**a**) grid antenna 1; (**b**) grid antenna 2; (**c**) grid antenna 3; (**d**) grid antenna 4.

**Figure 20:**
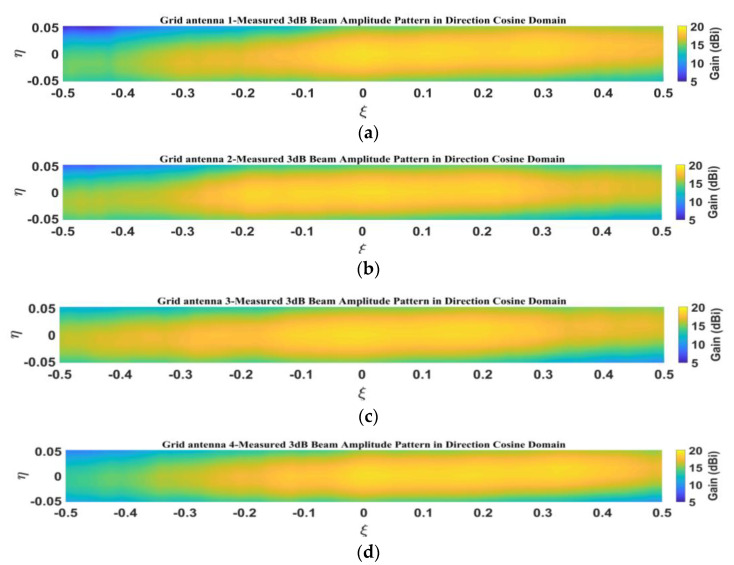
Measured amplitude pattern of 3-dB beamwidth in direction cosine domain: (**a**) grid antenna 1; (**b**) grid antenna 2; (**c**) grid antenna 3; (**d**) grid antenna 4.

Figure 19 presents the measured amplitude patterns of the four antenna elements for the sparse array in the directional cosine domain, and Figure 20 shows the measured amplitude patterns within the 3-dB beam range. From Figure 19 and Figure 20, it can be seen that the measured patterns of the four microstrip grid antennas are relatively consistent, and the amplitude patterns within the 3-dB beamwidth are in good agreement.

**Figure 21:**
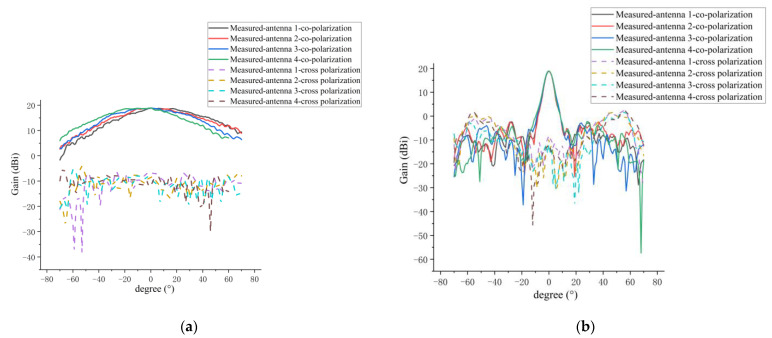
Measured amplitude pattern of 3-dB beamwidth for every antenna element in sparse arra:y (**a**) E-plane pattern; (**b**) H-plane pattern.

Figure 21a,b shows the measured co-polarization and cross-polarization patterns of every antenna element for the E and H planes in the 1D sparse array, respectively. A summary of the measured results is given in Table 5.

**Figure 22:**
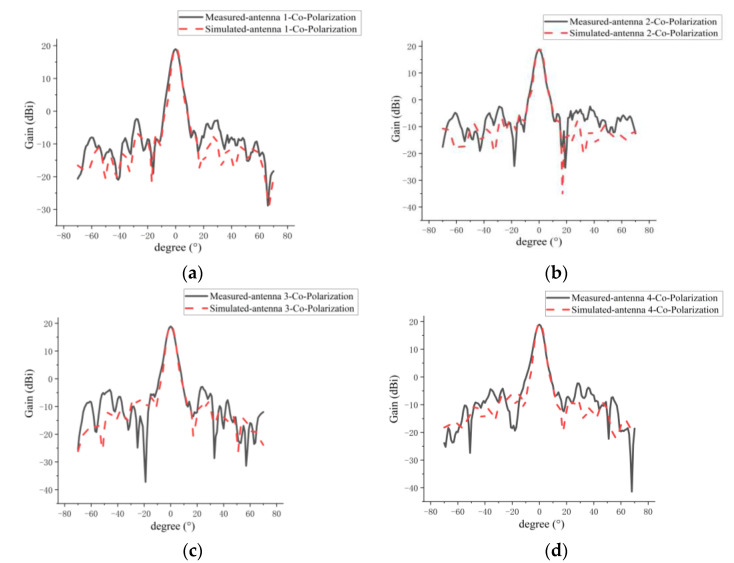
Comparison between measured and simulated H-plane co-polarization patterns of every antenna element in sparse array: (**a**) grid antenna 1; (**b**) grid antenna 2; (**c**) grid antenna 3; (**d**) grid antenna 4.

**Figure 23:**
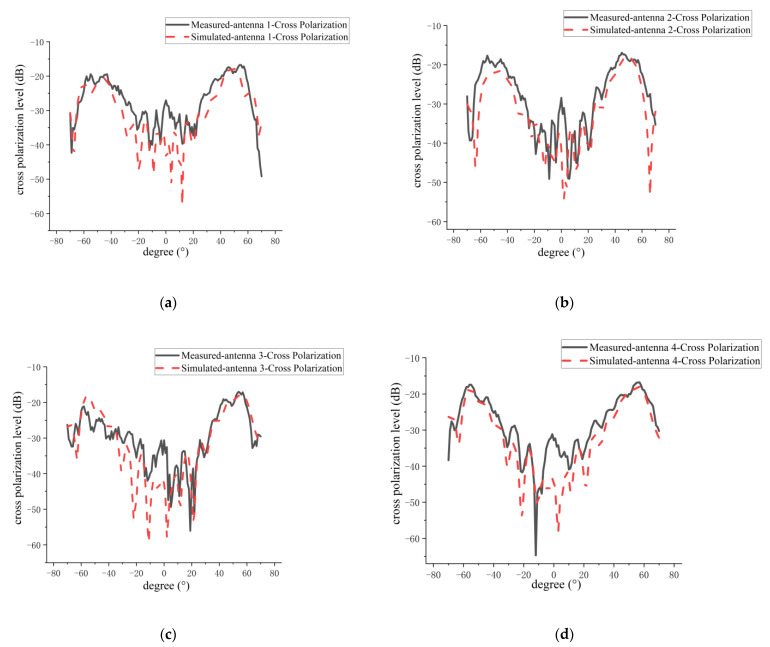
Comparison between measured and simulated H-plane cross-polarization patterns of every antenna element in sparse array: (**a**) grid antenna 1; (**b**) grid antenna 2; (**c**) grid antenna 3; (**d**) grid antenna 4.

The comparisons between the measured and simulated results of H-plane co-polarization and cross-polarization patterns for the four antenna elements in the 1D sparse array are shown in Figure 22 and Figure 23, respectively. A summary of the measured and simulated results is given in Table 6. As can be seen from Figure 22 and Figure 23 and Table 6, the measured and simulated results of gain are consistent, and the maximum difference is not more than 0.8 dB. The average of the measured sidelobe level of the four antennas is less than −20 dB. For the 3-dB beamwidth of the H-plane, the measured results of the 3-dB beamwidth are more consistent with the simulation, and the maximum difference is not more than 0.4°. The simulated and measured results of cross-polarization for the H plane are generally more consistent, and the maximum difference is about 1 dB. This 1 dB difference may be caused by the processing error of the antenna and the impact of the metal support structure for the sparse array. From Figure 22 and Figure 23, it can clearly be seen that the measured and simulated results of the H-plane patterns for the four elements in the sparse array are in good agreement.

**Figure 24:**
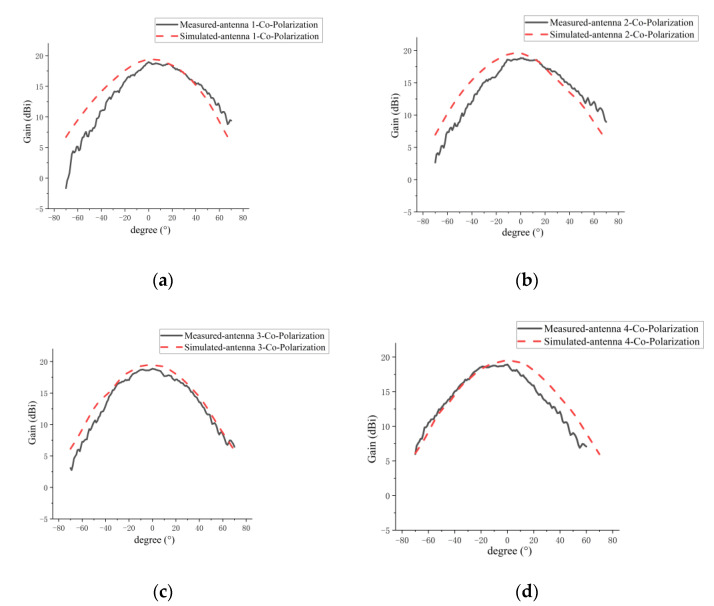
Comparison of measured and simulated E-plane co-polarization patterns for every antenna element in sparse array: (**a**) grid antenna 1; (**b**) grid antenna 2; (**c**) grid antenna 3; (**d**) grid antenna 4.

**Figure 25:**
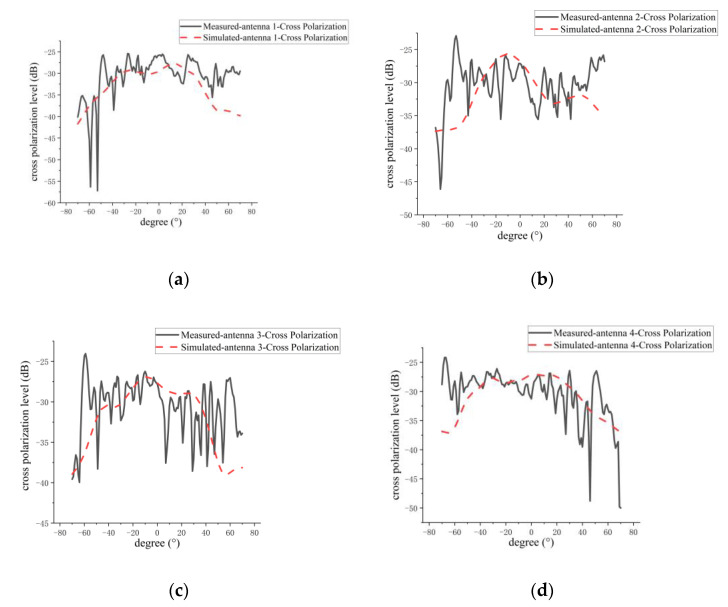
Comparison of measured and simulated E-plane cross-polarization patterns for every antenna element in sparse array: (**a**) grid antenna 1; (**b**) grid antenna 2; (**c**) grid antenna 3; (**d**) grid antenna 4.

Figure 24 and Figure 25 present a comparison between the measured and simulated results of the E-plane co-polarization and cross-polarization patterns for the four elements in the sparse array, respectively. The summary of the measured and simulated results is shown in Table 7. From Figure 24 and Table 7, it can be seen that the 3-dB beamwidth of the measured E-plane pattern is smaller than the simulated results, and the measured 3-dB beamwidth of the E plane for each antenna element in Table 7 becomes narrow compared with the E plane for the single grid antenna measurement in Table 4. An analysis of the causes is as follows. The fabrication error of the microstrip line can alter the current distribution, resulting in a 3-dB beamwidth reduction. Furthermore, coupling between elements in the array is responsible for the measured 3-dB beamwidth reduction in Table 7, and the coupling between elements of the fabricated antenna array has a greater effect on the 3-dB beamwidth in the E plane than in the simulated conditions. Moreover, compared with the H-plane pattern, the 3-dB beamwidth of the E-plane pattern for the microstrip grid antenna is wider, which makes it susceptible to coupling in the actual array, thus resulting in a narrower 3-dB beamwidth.

### 5.3. Measurement of Phase Patterns for 1D Non-Redundant Sparse Array

**Figure 26:**
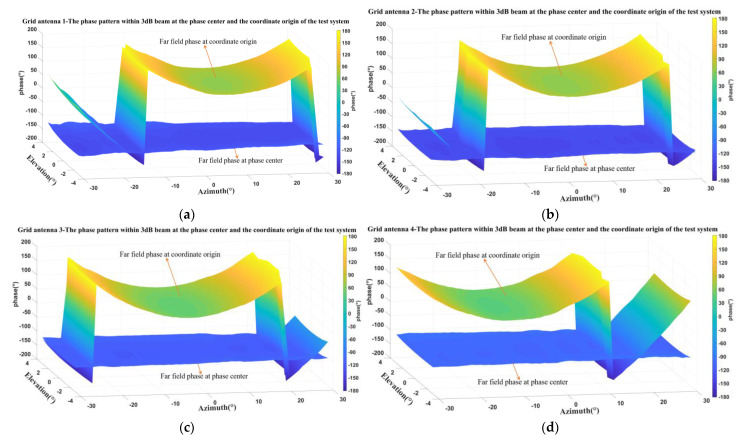
Measured far-field phase patterns at the coordinate origin and the phase center of every antenna element within the 3-dB beamwidth in sparse array: (**a**) grid antenna 1; (**b**) grid antenna 2; (**c**) grid antenna 3; (**d**) grid antenna 4.

The far-field phase pattern was still tested using the NSI2000 planar near-field measurement system. Figure 26 presents a comparison of the measured far-field phase pattern at the coordinate origin of the measurement system and the phase center within the 3-dB beamwidth for the four elements in the 1D non-redundant sparse array. It is obvious that the phase patterns at the coordinate origin have a large phase undulation, and the phase patterns at the phase center are much flatter.

**Figure 27:**
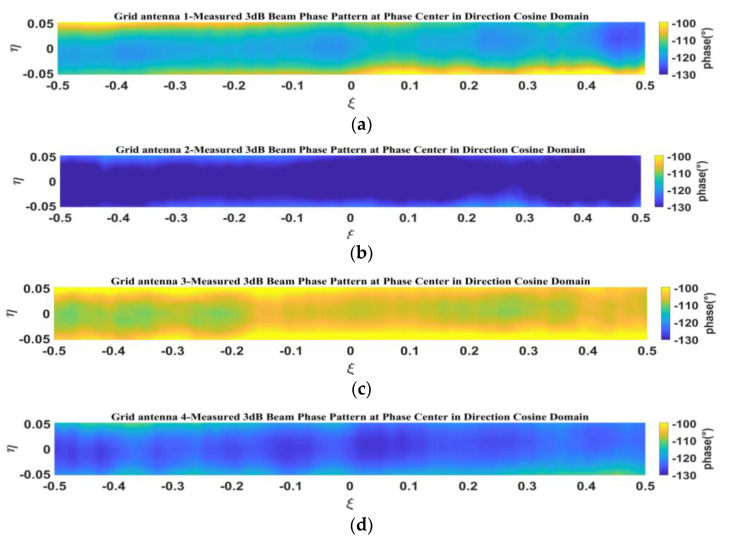
Measured phase pattern of 3-dB beamwidth at the phase center in direction cosine domain: (**a**) grid antenna 1; (**b**) grid antenna 2; (**c**) grid antenna 3; (**d**) grid antenna 4.

Figure 27 shows the measured phase pattern at the phase center within the 3-dB beamwidth in the direction cosine domain.

**Figure 28:**
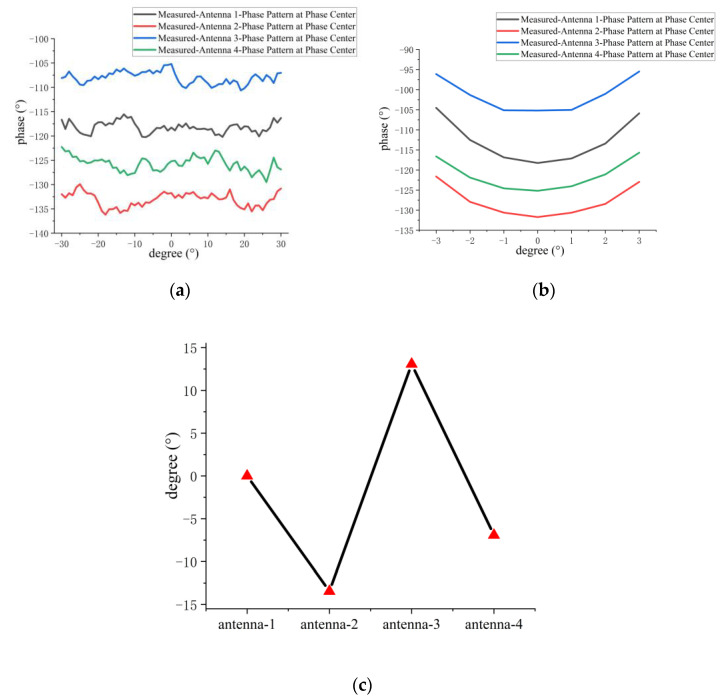
(**a**) Measured E-plane phase pattern of 3-dB beamwidth at the phase center for every antenna element in sparse array; (**b**) measured H-plane phase pattern of 3-dB beamwidth at the phase center for every antenna element in sparse array; (**c**) the phase difference of the remaining grid antennas relative to antenna 1, with the antenna beam pointing in the maximum radiation direction.

Figure 28a,b presents the measured phase patterns of four antenna elements at the phase center within the 3-dB beamwidth for the E and H planes, respectively. Figure 28c shows the phase difference of the remaining antennas relative to antenna 1 with the phase of antenna 1 as the reference phase (set to 0°) under the maximum radiation direction of the antenna. The measured and simulated phase fluctuations within the 3-dB beamwidth of the four elements in the sparse array are given in Table 8. From Figure 28a–c, it can be found that the measured phase patterns of the four microstrip grid antennas are in different phase ranges, which is due to the coupling between the antennas in the sparse array. From Table 8, it can be found that the difference between the measured and simulated phase fluctuations within the 3-dB beamwidth for the E plane is not more than 2.5° on average, and the H plane is about 3.4° on average. This indicates that the phase patterns of the microstrip grid antennas designed in the one-dimensional non-redundant sparse array are relatively stable.

A comparison between this work and the referenced designs is illustrated in Table 9. Among the reference designs in Table 9, refs. [17,18] are single-port microstrip grid antennas. The works in [19,22] include designs of a multi-port microstrip grid antenna with subarrays. It is obvious in Table 9 that the antenna proposed in this paper has a good sidelobe level and a low coupling performance for forming an array. Furthermore, the proposed fan-beam antenna has a narrower 3-dB beamwidth of the narrow beam and a wider 3-dB beamwidth of the wide beam, with a sufficiently small antenna width.

## 6. Conclusions

In this paper, a kind of fan-beam microstrip grid antenna is designed for a K-band 1D MIR airborne prototype of a MICAP. This paper proposes the periodic structural unit for designing the microstrip grid antenna. By using the periodic structural units instead of all radiation cells for Taylor weighting, the SLL of the H-plane pattern (narrow beam direction of the fan beam) is less than −20 dB, and the design complexity of the antenna is reduced. In addition, using the periodic structural unit ensures that the antenna has sufficient radiating cells to make the antenna gain up to 19 dB and also to produce a 3-dB beamwidth in the narrow beam direction (H plane) of 5.34° to ensure the resolution of the 1D MIR along the track direction. Furthermore, the width of the antenna designed in this paper is very narrow (0.77λ). Under this condition, the antenna has 3-dB beamwidth of up to 55° in the wide beam direction (E-plane). The performance of the one-dimensional non-redundant sparse array formed by the proposed antenna is analyzed. It is found that in the array environment, the coupling between antenna elements is less than −25 dB, and the consistency of the amplitude patterns for every element is good. The average difference between the phase fluctuations of the measured and simulated phase patterns in the E and H planes is not more than 3.4°. These results verify that the microstrip grid antenna proposed in this paper can completely meet the requirements of a K-band 1D MIR airborne prototype of a MICAP.

## Figures and Tables

**Table 1:** Geometric parameters of grid antenna.

Name	l	s	wl	ws1	ws2	ws3	ws4	ws5	ws6
Size (mm)	12.6	5.43	0.2	0.1	0.3	0.9	1.5	1.9	2.2

**Table 2:** Theoretical normalized current values calculated by Taylor weighting and simulated normalized current values for 11 periodic structural units.

Periodic Structural Unit	Theoretical Normalized Current Value	Simulated Normalized Current Value
1	0.5234	0.1954
2	0.6013	0.2686
3	0.7231	0.5643
4	0.8401	0.8102
5	0.9605	0.9282
6	1	1

**Table 3:** Simulated phase ranges and fluctuations of 3-dB beamwidth phase patterns in E and H planes for every antenna element.

	E-Plane Phase Range within 3-dB Beamwidth	E-Plane Phase Fluctuation	H-Plane Phase Range within 3-dB Beamwidth	H-Plane Phase Fluctuation
Grid antenna 1	12.9–17.4°	4.5°	12.9–20.8°	7.9°
Grid antenna 2	7.8–15.1°	7.3°	9.5–17.9°	8.4°
Grid antenna 3	18.5–21°	2.5°	20.5–27.2°	6.7°
Grid antenna 4	19–20.5°	1.5°	21–27.3°	6.3°

**Table 4:** Comparison of simulation and measurement of microstrip grid antenna.

	Simulation Results	Measured Results
Gain	19.4 dBi	19.1 dBi
Half-power beamwidth of E plane	61°	59.91°
Cross-polarization of E plane	<−28 dB	<−26 dB
Half-power beamwidth of H plane	4.7°	5.34°
H-plane sidelobe level	<−25 dB	<−21.4 dB
Cross-polarization of H plane	<−18.7 dB	<−17.37 dB
VSWR	frequency band < 1.53 center frequency: 1.12	frequency band < 1.82 center frequency: 1.56

**Table 5:** Measured results of E- and H-plane patterns for every antenna element in sparse array.

	Gain (dBi)	E-Plane Cross-Polarization (dB)	E-Plane 3-dB Beamwidth (°)	H-Plane Sidelobe Level (dB)	H-Plane Cross-Polarization (dB)	H-Plane 3-dB Beamwidth (°)
Grid antenna 1	18.93	−25.2	54.6	−21.17	−16.71	5.25
Grid antenna 2	18.83	−23	53.85	−20.1	−16.93	4.97
Grid antenna 3	18.87	−24	57.69	−21.45	−17	5.24
Grid antenna 4	18.9	−24.17	56.55	−21.22	−16.76	5.25

**Table 6:** Measured and simulated results of H-plane patterns for every antenna element in sparse array.

	Measured Gain (dBi)	Simulated Gain (dBi)	Measured H-Plane Sidelobe Level (dB)	Simulated H-Plane Sidelobe Level (dB)	Measured H-Plane Cross-Polarization (dB)	Simulated H-Plane Cross-Polarization (dB)	Measured H-Plane 3-dB Beamwidth (°)	Simulated H-Plane 3-dB Beamwidth (°)
Grid antenna 1	18.93	19.34	−21.17	−26.35	−16.71	−17.78	5.25	4.84
Grid antenna 2	18.83	19.55	−20.1	−24.58	−16.93	−18	4.97	4.73
Grid antenna 3	18.87	19.38	−21.45	−25.4	−17	−17.69	5.24	4.95
Grid antenna 4	18.9	19.53	−21.22	−25.52	−16.76	−17.9	5.25	4.95

**Table 7:** Measured and simulated results of E-plane patterns for every antenna element in sparse array.

	Measured E-Plane Cross-Polarization (dB)	Simulated E-Plane Cross-Polarization (dB)	Measured E-Plane 3-dB Beamwidth (°)	Simulated E-Plane 3-dB Beamwidth (°)
Grid antenna 1	−25.2	−27.77	54.6	62.07
Grid antenna 2	−23	−25.67	53.85	56.92
Grid antenna 3	−24	−26.9	57.69	61
Grid antenna 4	−24.17	−27.1	56.55	60

**Table 8:** Measured and simulated E- and H-plane phase fluctuations within 3-dB beamwidth of every antenna element in the sparse array.

	Measured E-Plane Phase Fluctuation within 3-dB Beam	Simulated E-Plane Phase Fluctuation within 3-dB Beam	Measured H-Plane Phase Fluctuation within 3-dB Beam	Simulated H-Plane Phase Fluctuation within 3-dB Beam
Grid antenna 1	4.7°	4.5°	13.73°	7.9°
Grid antenna 2	6.26°	7.3°	10.1°	8.4°
Grid antenna 3	5.43°	2.5°	9.71°	6.7°
Grid antenna 4	7.24°	1.5°	9.5°	6.3°

**Table 9:** Performance comparison of the microstrip grid antennas around K band.

References	Working Frequency (GHz)	Sidelobe Level (dB)	HPBW	Gain (dB)	Antenna Size	Coupling (dB)
[17]	24	−16	7° (narrow beam) 90° (wide beam)	13.87	1.44λ × 11.68λ	—
[18]	24	−16	16°	19.26	4.8λ × 4.8λ	—
[19]	24	−15	14°	20.6	4.8λ × 4.8λ	−17.5 dB
[22]	24	−15	—	22.5	8λ × 8λ	−34 dB
This work	18.7	−21.4	5.34° (narrow beam) 55° (wide beam)	19.1	0.77λ × 13.3λ	−25 dB

## Data Availability

Not applicable.
